# Multi-slice representational learning of convolutional neural network for Alzheimer’s disease classification using positron emission tomography

**DOI:** 10.1186/s12938-020-00813-z

**Published:** 2020-09-07

**Authors:** Han Woong Kim, Ha Eun Lee, KyeongTaek Oh, Sangwon Lee, Mijin Yun, Sun K. Yoo

**Affiliations:** 1 Department of Medical Engineering, Yonsei University College of Medicine, Seoul, Republic of Korea; 2 Department of Nuclear Medicine, Yonsei University College of Medicine, Seoul, Republic of Korea

**Keywords:** Alzheimer’s disease, F-18 FDG-PET/CT, Deep learning, Convolutional neural network, External validation, Feasibility study

## Abstract

**Background:**

Alzheimer’s Disease (AD) is a degenerative brain disorder that often occurs in people over 65 years old. As advanced AD is difficult to manage, accurate diagnosis of the disorder is critical. Previous studies have revealed effective deep learning methods of classification. However, deep learning methods require a large number of image datasets. Moreover, medical images are affected by various environmental factors. In the current study, we propose a deep learning-based method for diagnosis of Alzheimer’s disease (AD) that is less sensitive to different datasets for external validation, based upon F-18 fluorodeoxyglucose positron emission tomography/computed tomography (FDG-PET/CT).

**Results:**

The accuracy, sensitivity, and specificity of our proposed network were 86.09%, 80.00%, and 92.96% (respectively) using our dataset, and 91.02%, 87.93%, and 93.57% (respectively) using the Alzheimer’s Disease Neuroimaging Initiative (ADNI) dataset. We observed that our model classified AD and normal cognitive (NC) cases based on the posterior cingulate cortex (PCC), where pathological changes occur in AD. The performance of the GAP layer was considered statistically significant compared to the fully connected layer in both datasets for accuracy, sensitivity, and specificity (*p* < 0.01). In addition, performance comparison between the ADNI dataset and our dataset showed no statistically significant differences in accuracy, sensitivity, and specificity (*p* > 0.05).

**Conclusions:**

The proposed model demonstrated the effectiveness of AD classification using the GAP layer. Our model learned the AD features from PCC in both the ADNI and Severance datasets, which can be seen in the heatmap. Furthermore, we showed that there were no significant differences in performance using statistical analysis.

## Background

Alzheimer’s Disease (AD), characterized by a decline in cognitive function, is one of the most common degenerative brain disorders. Because this disease characteristically presents in people over 65 years old, the incidence of AD has increased sharply in concert with the increase of the elderly population. Given the difficulties in managing the advanced stages of AD, accurate diagnosis of the disorder is important for effective treatment [1]. F-18 fluorodeoxyglucose (FDG) positron emission tomography/computed tomography (PET/CT) has been widely used to diagnose AD by comparing the rate of glucose metabolism throughout the brain [2].

A convolutional neural network (CNN) is a highly effective method of deep learning used to analyze and classify visual images of all kinds that cannot be resolved by conventional machine learning algorithms. An error rate of 3.5% has been demonstrated with the use of CNN [3], a value less than the error rate of manual classification by humans in classification of the CIFAR10 dataset [4]. Krizhevsky et al. [5] reported an error rate of 15.3% at the Image-net Large-Scale Visual Recognition Competition (ILSVRC), significantly lower than the 26.2% error rate reported using the conventional machine learning method. Because the CNN-based method extracts appropriate features learned from images, unlike conventional machine learning algorithms, it does not require domain knowledge to extract Regions of Interest (ROI) or handcrafted features.

Deep learning methods have recently been applied to AD classification. An autoencoder was applied to extract high-level features from MRI and PET images [6]. Suk et al. [7] built a robust model using a stacked autoencoder to extract and fuse features from MRI, PET, and cerebrospinal fluid (CSF) images. CNN also successfully achieved successful results in AD classification. Wang et al. [8] improved performance using leaky ReLU and max pooling in a CNN model. Basheera et al. [9] extracted gray matter, which changes its texture when affected by AD. The authors then used those segmentations as input data. Choi et al. [10] proposed a CNN method using segmented hippocampus ROI for anatomical information. Many studies have applied 3D CNN to take advantage of whole volume data. Feng et al. [11] used 3D CNN to obtain spatial information in feature maps. Huang et al. [12] integrated T1-weighted MR and FDG-PET in hippocampus ROI as 3D CNN input. Liu et al. [13] used 3D CNN to extract high-level features from MRI and PET, and then cascaded 2D CNN to combine features for AD classification.

Although these studies achieved successful results, limitations still exist. While gathering large amounts of imaging modality and numerical data may enhance performance, it is difficult to collect such data owing to limitations of of time, cost, and privacy. Moreover, additional pre-process methods, such as segmentation, require precise professional knowledge. An advantage of 3D CNN is that the model enables the extraction of voxel information. However, the model requires a number of image datasets for training, which is a significant problem in the medical imaging field. Moreover, the previous studies have not compared the results to other test datasets. Medical images are heavily affected by the imaging acquisition environment, acquisition instrument, protocols, and reconstruction method [14]. Therefore, it is difficult to train models with open datasets since the models tend to perform poorly when models are evaluated with external test datasets. Advantages and disadvantages for each method are summarized in Table 1.

**Table 1:** Comparison of AD classification methods

Author (year)	Image modality	Pre-processing	Method	Advantage	Disadvantage
Liu et al. [6]	MRI, PET	Feature extraction & selection	Autoencoder	Extracted high-level features	Difficulties in gathering various imaging modality and numerical data
Suk et al. [7]	MRI, PET, CSF	Gray matter/white matter segmentation, feature extraction	Stacked autoencoder	Extracted and fused high-level features	
Basheera et al. [9]	MRI	Gray matter segmentation	CNN	Focused on gray matter features	Require a precise professional knowledge
Choi et al. [10]	MRI	Hippocampus segmentation	CNN	Improved performance using small patches as input	
Wang et al. [8]	MRI	Spatial and intensity normalization	CNN + RELU + max pooling	Improved performance of CNN	Requires evaluation with different image acquisition environment dataset
Feng et al. [11]	MRI, PET	Gray matter segmentation	3D CNN + LSTM	Obtained spatial information	3D model requires a number of image datasets for training
Huang et al. [12]	T1-MR, FDG-PET	Hippocampus segmentation	3D CNN	Integrated T1 weighted MR and FDG-PET as input	
Liu et al. [13]	MRI PET	Spatial and intensity normalization	3D CNN + cascaded 2D CNN	Extracted multi-level and multi-modal features	

Based on the previous considerations, we applied the CNN deep learning method to classify AD and normal cognitive (NC) cases in a manner less sensitive to the image acquisition environment. Our network showed feasibility in training and testing using different FDG-PET/CT datasets. We trained our model with the Alzheimer’s Disease Neuroimaging Initiative (ADNI) dataset and tested it with the Severance dataset. In addition, we applied global averaging pooling (GAP), so that our model can provide a heatmap to visualize which region is relatively important for classification.

The study was designed in the following way. The Methods section describes the proposed approach in detail, as follows: description of both the training and test datasets, pre-processing methods for intensity and spatial normalization, detailed methods used to classify AD based on CNN, how the model was trained based on loss function, and statistical evaluation to analyze the results. In the Results section, we demonstrated classification performances. First, we highlighted GAP by comparing with the fully connected layer. In addition, we analyzed the generalization performance with statistical evaluation. The results indicated that our proposed method did not show statistical differences on datasets attained from different image acquisition environment. The Discussion section mentions implications from our findings that our model noticed the anatomical changes as clinicians do in practice. The Conclusion section summarizes our results.

## Results

### Classification results

**Fig. 1:**
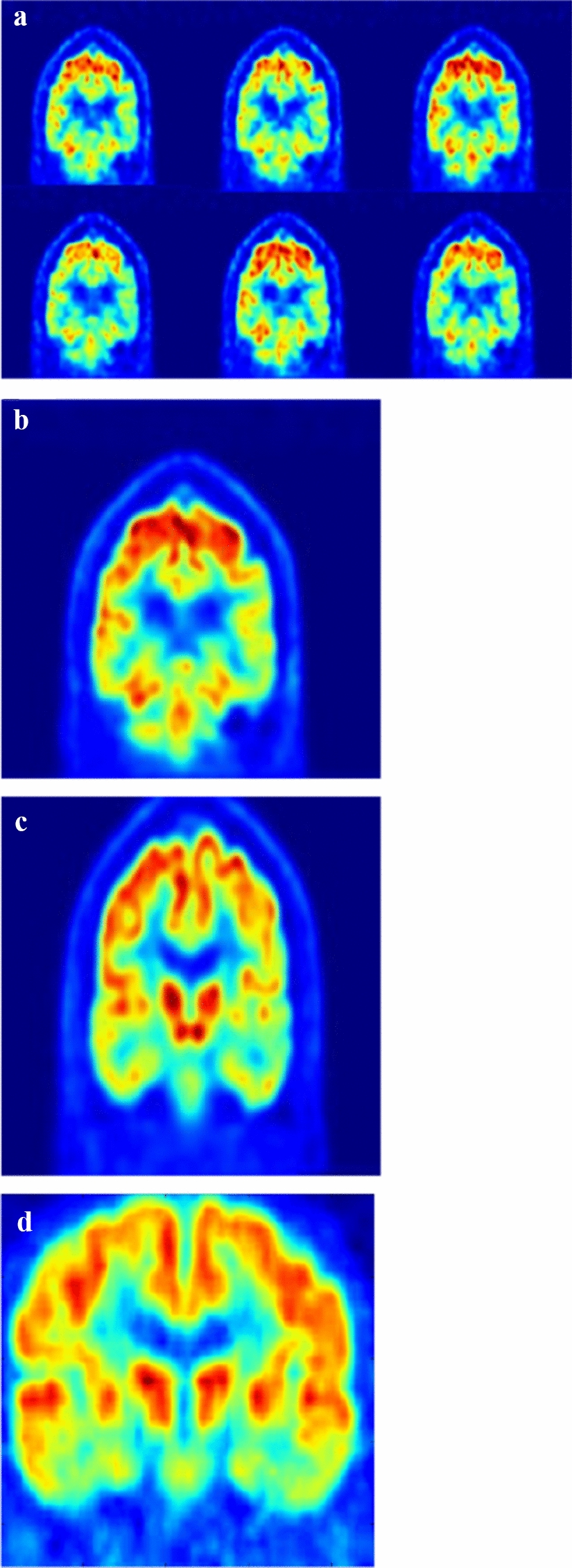
An overview of FDG-PET pre-processing **a** Co-registered dynamic images of PET images. **b** Averaged PET image. **c** Intensity normalized PET image **d** Spatial normalized PET image

**Fig. 2:**
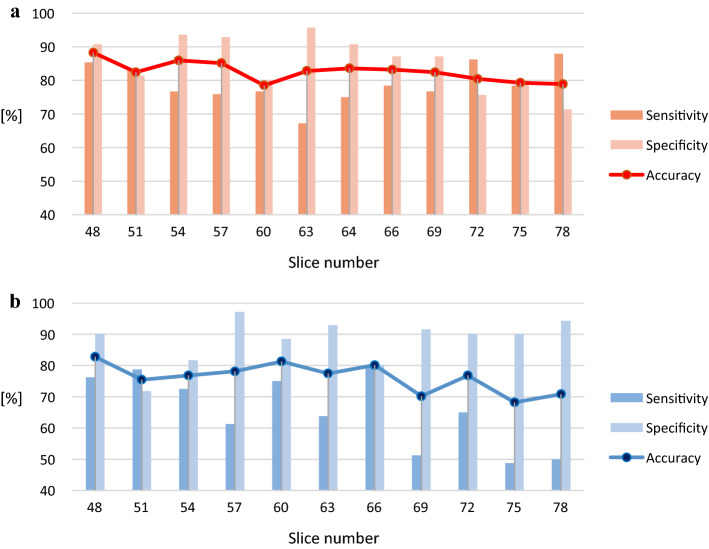
Classification results of single-input network depending on the slice numbers **a** using ADNI dataset and **b** using our dataset

**Fig. 3:**
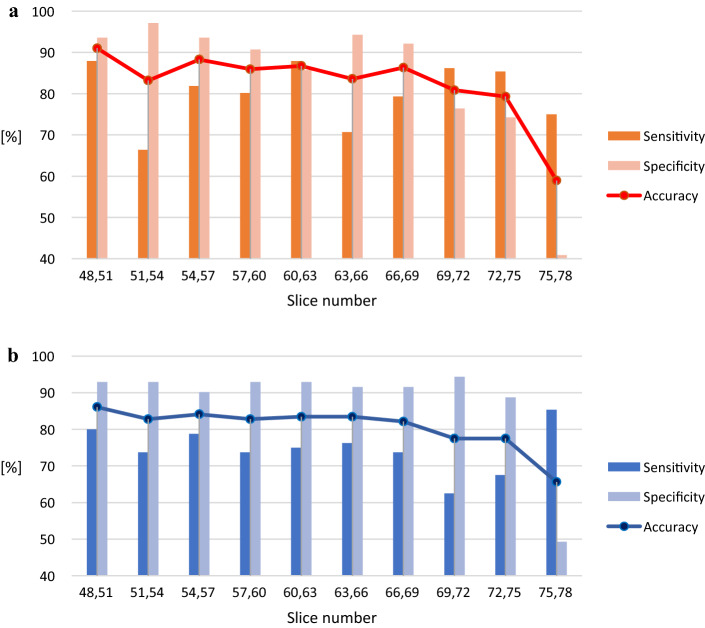
Classification results of double inputs network depending on the slice numbers **a** using ADNI dataset and **b** using our dataset

Figure 1 illustrates pre-processed FDG-PET/CT scans. As mentioned, the ranges of slice numbers were chosen to include those that cover the locations where neuropathological changes occur in AD. To compare the performance, accuracy, sensitivity, and specificity were calculated. The results of single input are shown in Fig. 2. Within the slice range, the best results of accuracy, sensitivity, and specificity with the ADNI data were 88.28%, 85.34%, and 90.17% (respectively), and those with our dataset were 82.78%, 76.25%, and 90.14%, respectively. The results of double inputs are shown in Fig. 3. The results for accuracy, sensitivity, and specificity with the ADNI data were 91.02%, 87.93%, and 93.57% (respectively), and those with our dataset were 86.09%, 80.00%, and 92.96%, respectively. The proposed network showed improvement in all performance measures. Accuracy, sensitivity, and specificity were enhanced by 3.34%, 3.75%, and 2.82% with the ADNI dataset and by 2.74%, 2.59%, and 2.86% with our dataset.

### Global average pooling performance

To evaluate the performance of the GAP layer in the network, we compared the results to a network with and without the GAP layer. This is because our model replaced a fully connected layer to the GAP layer at the end of the layer to improve performance. Thus, a fully connected network (FCN) represents the architecture published by Krizhevsky et al. [5], whereas our model applied and changed based on the architecture. As shown in Table 2, the network with the GAP layer showed better performance in all measures. Using our dataset, the proposed network showed accuracy, sensitivity, and specificity of 86.09%, 80.00%, and 92.96%, respectively, representing improvements of 11% and 20% in accuracy and sensitivity compared to an FCN. Using the ADNI data, our proposed network showed accuracy, sensitivity, and specificity of 91.02%, 87.93%, and 93.57%, respectively (improvements of 15%, 5%, and 21%, respectively, compared to FCN). The *p* value was calculated using McNemar’s test. The *p* value indicates that the difference in accuracy between the network with and without GAP layers is considered statistically significant in each dataset (*p* < 0.05).

**Table 2:** Classification results comparison between FCN and GAP network (proposed) with *p* value

Dataset	Model	ACC [%]	SENS [%]	SPEC [%]	*p* value
Our dataset	FCN	75.50	60.00	92.96	*p* < 0.001
	GAP (Proposed)	86.09	80.00	92.96	
ADNI	FCN	76.95	82.76	72.14	*p* < 0.01
	GAP (Proposed)	91.02	87.93	93.57	

*FCN* fully connected network, *GAP* global average pooling

### Generalization performance

Another *p* value was calculated to validate that our network has consistent performance with ADNI data (not used in training) and with our institutional data. For statistical comparison of their performance, a *p* value was obtained using Pearson’s Chi-square test. Table 3 summarizes the *p* values for accuracy, sensitivity, and specificity. All *p* values between ADNI and our dataset performance are considered not significant. Accordingly, the *p* values indicate that our network is less sensitive to test datasets (*p* > 0.05).

**Table 3:** Classification results comparison between ADNI and our dataset with *p* value

Performance	Our dataset	ADNI	*p* value
ACC [%]	86.09	91.02	0.17 (n.s)
SENS [%]	80.00	87.93	0.19 (n.s)
SPEC [%]	92.96	93.57	0.44 (n.s)

*n.s* not significant, *ACC* accuracy*, SENS* sensitivity*, SPEC* specificity

**Fig. 4:**
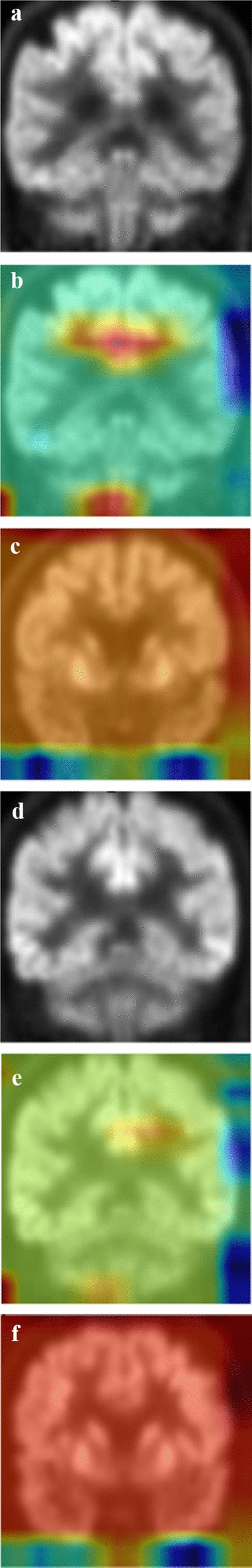
Selected pre-processed FDG-PET input image of AD patient **a** heatmap of the corresponding input with the best accuracy **b** and the worst accuracy. **c** Selected pre-processed FDG-PET input image of NC. **d** Heatmap of the corresponding input with the best accuracy **e** and the worst accuracy **f**

Moreover, we observed that the heatmap showed different highlighted regions depending on slice inputs. To compare the heatmap of each slice, Fig. 4 illustrates the heatmaps with the best and the worst accuracy in the network. The network with the best accuracy noticed the posterior cingulate cortex, where the metabolic reduction occurring in the early stages of AD [15] decreased. On the other hand, the network with the worst accuracy showed that the network learned inappropriate features outside of the brain.

To compare the generalization capability, we observed the results using other AD classification methods, shown in Table 4. We compared the results using slice numbers that were closest to our model. The training for each iteration step required 5 s for He et al. [16] and 6 s for Huang et al. [17]. The results showed that other AD classification methods showed similar performance using the ADNI dataset. However, AD classification methods showed lower performance using our dataset, which has a different image acquisition environment. This finding demonstrates that our model has improved generalization capability.

**Table 4:** Alzheimer’s disease classification performance using our methods and other AD classification models

	ADNI			Our dataset		
	ACC [%]	SENS [%]	SPEC [%]	ACC [%]	SENS [%]	SPEC [%]
He et al. [17]	90.94	85.53	96.18	81.56	71.23	92.65
Huang et al. [18]	91.26	84.21	98.09	82.98	72.60	94.12
Our model	91.02	87.93	93.57	86.09	80.00	92.96

*ACC* accuracy*, SENS* sensitivity*, SPEC* specificity

## Discussion

Recently, deep learning has been applied to achieve state-of-the-art classification results in the fields of pattern recognition, human voice, image processing, and medical imaging. In the field of neuroimaging, the prediction accuracy of classification using deep learning has improved [18, 19]. In this study, we proposed a deep learning network that can diagnose patients with AD, and is less sensitive to institutional FDG-PET/CT datasets using different protocols.

We applied the transfer learning method to extract features accurately from 1245 FDG-PET/CT images. In general, CNN requires more data and more time to train complex models [5]. Transfer learning is a deep learning technique to fine-tune a model that is already trained with a large number of data images. This technique may reduce the difficulties in training complex models of setting parameters such as the number of layers, activation function, and hyperparameters. Transfer learning enabled the network to learn with a smaller number of FDG-PET/CT images.

To enhance general performance, we applied the GAP layer instead of the fully connected layer to our network. One of the advantages of using a GAP layer is object localization. With use of the GAP layer in our CNN, the network provides explanation to users by showing a heatmap. These heatmaps indicate that it is significant to choose appropriate slice numbers, because the network considers different areas important. It appears that the areas of the network identified were also clinically related to AD classification. Thus, we expected our network to recognize the entorhinal cortex or hippocampus located in the mesial temporal lobe where neuropathological changes occur in the beginning stages of AD. However, the mesial temporal lobe is sensitive and contains small amounts of information that may be negatively affected by the partial volume effect (PVE) [20] during the pre-processing process. We assume that the PVE made it difficult for the network to detect the mesial temporal lobe, since the limited spatial resolution of PET scanners caused deteriorated in the quality of PET images.

Moreover, we found that the network identifies different heatmap areas depending on the input slice. For more sophisticated classification, our network used two consecutive slice inputs. We first trained the single-input network with two subsequent slices individually; then, we added a fully connected layer to combine those results. We assumed that the network using single input would have insufficient information for diagnosing AD. Different indicated areas depending on the input slice number may have contributed to performance enhancement. Our proposed network performance exceeded all measurements of the single-input network by an average of 3%.

The work detailed in this report has been illuminating; however, our findings require further study. This research relied on a coronal plane, as neuropathologic changes on autopsy are assessed using coronal slices. Experiments in the axial and sagittal planes are also required for further research. Also, it is necessary to verify that consistent performance is valid with various institution datasets. Because medical datasets are difficult to acquire and are expensive, we used only our own dataset to determine the generalization performance of the proposed network. In addition, further study and experiments with architectures that could include more inputs are needed to observe the network improvements.

For further study, we consider applying our method to other critical diseases that show anatomical changes as the disease progresses. Since our model provides clinical information through a heatmap, our model is appropriate for application to other classification problems in cases that are diagnosed with specific scans. Aside from neurological data, our model may be used for other diseases in further study. For example, Lyssek-Boroń et al. [21] demonstrated that Epiretinal Membrane (ERM) is diagnosed with Optical Coherence Tomography (OCT) by Retinal Nerve Fiber Layer (RNFL) thickness. Krysik et al. [22] analyzed the central and peripheral corneal thickness using Pentacam Scheimpflug camera and OCT. In addition, we can yield heatmaps as quantitative risk maps using MRI for prostate cancer [23]. We will work to expand the applications of our model.

## Conclusions

In the present study, we demonstrated that our network performed consistently by training our model with the ADNI dataset and testing it with our dataset. With insufficient datasets, we applied slice selective learning to reduce computational costs. We also statistically enhanced the generalization performance by applying transfer learning and the GAP layer. Our CNN-based method showed the feasibility of robustness to institutional datasets when automatically classifying subjects with NC and AD.

## Methods

### Dataset

For training and validation data, we used FDG-PET/CT data from the Alzheimer’s Disease Neuroimaging Initiative (ADNI), which includes 141 AD patients and 348 NC participants. The demographics of the ADNI dataset are shown in Table 5a. A dose of FDG (185 MBq; 5 mCi) was injected into subjects. ADNI FDG-PET/CT images of six 5-min frames were obtained 30–60 min after the injection. The FDG-PET/CT images were reoriented into standardized 1.5 × 1.5 × 1.5 mm voxel size.

**Table 5:** Demographic description of the ADNI dataset

Diagnosis	Number	Age (avg ± std)	Sex (M/F)
(a) ADNI dataset			
AD	141	75.92 ± 7.9	92/49
NC	348	76.28 ± 6.4	173/175
(b) Severance dataset			
AD	80	71.05 ± 9.3	50/30
NC	72	63.33 ± 9.3	33/39

*AD* Alzheimer’s Disease*, NC* normal cognitive, avg average, std standard deviation

For the tests, our FDG-PET/CT data included 71 NC participants and 80 AD patients using our own FDG-PET/CT acquisition process based on a cohort study. Our FDG-PET/CT data collection was approved by the Institutional Review Board (4-2018-1010). The demographics are shown in Table 5b. FDG-PET/CT images were acquired using a Discovery 600 (GE Medical Systems, Milwaukee, WI, USA) PET/CT scanner in the Nuclear Medicine Department of Severance Hospital (Seoul, South Korea). Approximately 4.1 MBq of 18F-FDG per kilogram of body weight was administered intravenously to the subjects. Forty minutes after 18F-FDG injection, PET images were acquired for 15 min. Spiral CT scans were performed for attenuation correction with 0.8 s rotation time, 60 mA, 120 kVp, 3.75 mm section thickness, 0.625 mm collimation, and 9.375 mm table feed per rotation. We reconstructed the FDG-PET/CT images using the ordered subset expectation maximization algorithm (4 iterations and 32 subsets).

### Pre-processing

First, we processed the raw FDG-PET scans using the method described by Jagust et al. [24]. Each FDG-PET scan was co-registered to the first frame of the raw FDG-PET scan to reduce the effects of subject motion. We generated a single PET scan by averaging six dynamic frames, and then reoriented the co-registered and averaged scan into a standard voxel image grid with 1.5 mm 3 voxels.

We normalized the voxel intensity of processed FDG-PET scans using an iterative method previously described. For the first iteration, the entire image was scaled to a mean intensity value of 1.0. Successive iterations masked voxels with intensity values lower than 0.5. We rescaled the remaining voxels to a mean of 1.0. Equation 1 illustrates the intensity normalization in a step. Mean is the average value of whole volume, and i describes each pixel. The voxel intensity of the FDG-PET was normalized by repeating this process until the number of remaining voxels became constant:

1 $$ {\text{rescaled volume}}\left[ i \right] = \left\{ {\begin{array}{*{20}c} {\frac{{{\text{volume}}\left[ i \right]}}{\text{mean}}  {\text{if volume}}\left[ i \right] > {\text{mean}}} \\ {{\text{volume}}\left[ i \right]} \\ \end{array}  } \right.. $$

After intensity normalization, each FDG-PET scan displayed the difference between a subject’s brain size and shape. Thus, the same brain regions appeared in the same position in all the patients’ brain scans. We use a spatial normalization method based on the MNI-152 template [25].

### Slice selection

Owing to the lack of sufficient data, we reduced the computational cost using slice selective learning. Each coronal 2D slice has 1.5 mm thickness and we extracted at 4.5 mm intervals from the FDG-PET/CT data. Generally, coronal slices are cut at 5–7.5 mm for metastases, infarcts, etc. in brain autopsies [26]. The extracted slices were numbered from the back of the head. The slice range was included in the regions where the neuropathological change takes place in AD, such as the posterior cingulate cortex, hippocampus, and entorhinal cortex [15, 27]. Our network was trained with two coronal slices for additional volume information.

### Network architecture

**Fig. 5:**
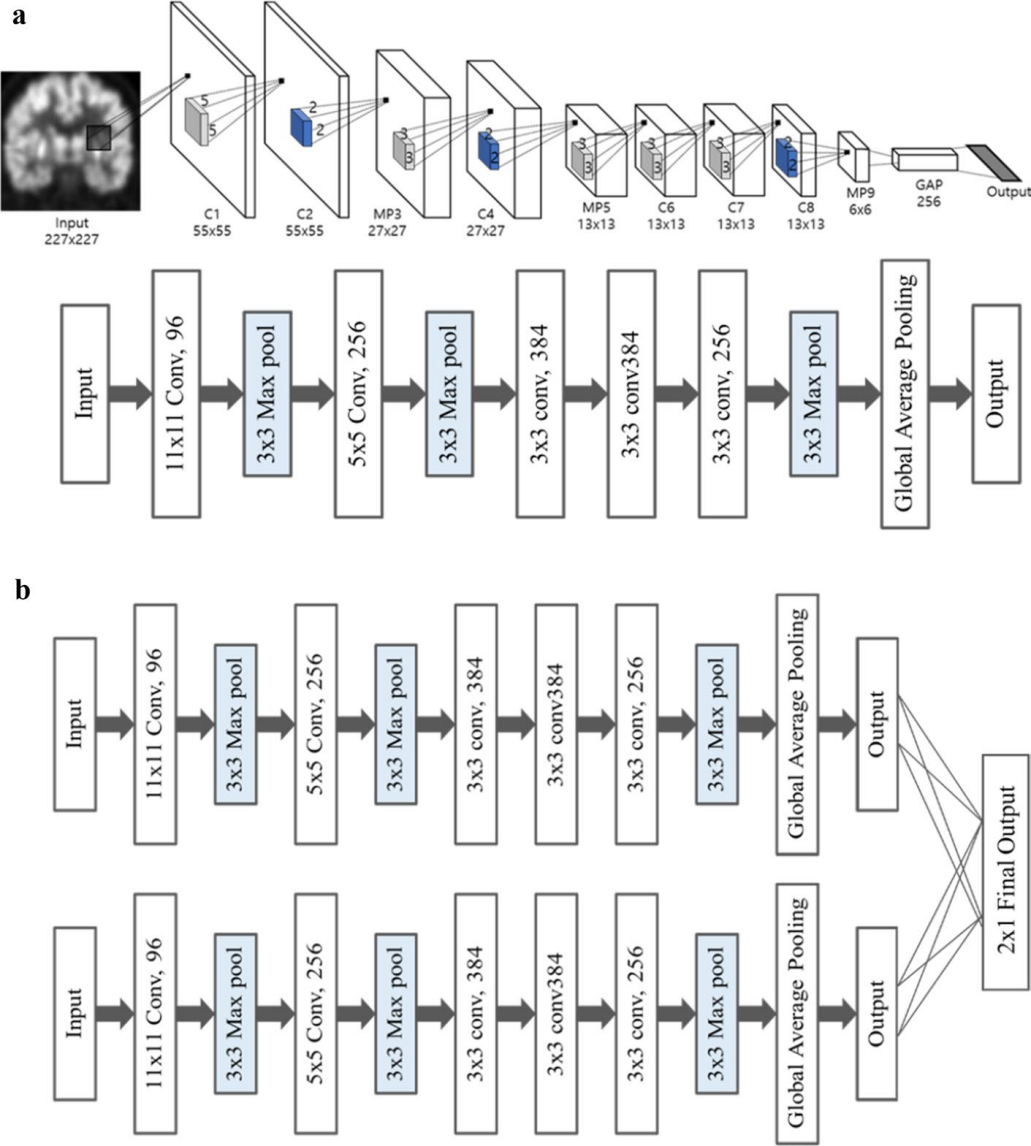
Overview of the proposed method **a** The network of one input architecture for classifying mild Alzheimer’s Disease, **b** Our proposed architecture of convolutional neural network for classifying mild Alzheimer’s Disease

We used the CNN-based method to classify AD and NC without extracting handcrafted features and to learn generic features from FDG-PET images. The proposed network is based on architecture published by Krizhevsky et al. [5], as shown in Fig. 5. The proposed network consists of ten convolutional layers, six max pooling layers, two GAP layers, and three fully connected layers. We replaced the last fully connected layer with a GAP layer, as illustrated in Fig. 5a. A fully connected layer was used as the last layer of our model to combine the double slice input, as illustrated in Fig. 5b.

Conventional CNN generally extracts features from the input image using the convolutional layer. Then, the fully connected layer is trained to classify images based on the extracted features. However, the fully connected layer is prone to overfitting owing to the large number of parameters that are required to be trained. This factor leads to decreased generalization performance of the entire network [28].

Thus, we applied a GAP layer instead of a fully connected layer. The GAP layer takes an average of each feature map generated from feature extraction. Because fewer parameters are needed for training compared to a network without a GAP layer, it is less likely to be overfitted. Moreover, the GAP layer embeds global contextual information [29]. We can obtain a heatmap of input that shows which region is relatively important for making a classification decision.

### Training and evaluation

The model was trained with Adam optimizer with a default learning rate parameter of 0.001. We reduced the learning rate by a decay factor of 0.25 for every 30 epochs. We set an epoch size of 4000 and batch size of 64. In addition, we set a dropout to 0.8 for overfitting. The training for each iteration step required 4 s. We determined the parameters based on the performance through training experiments. For loss function, binary cross-entropy function was used as indicated in Eq. 1. In each training step, the loss was used to update the parameters, where t is a target label and f(s) is the score at the softmax layer of the input data. In the softmax layer, the model predicts the probability of AD. If the target value is 0, which is NC, the loss will drastically increase when the score becomes close to 1, whereas the loss is 0 when the model predicted 0. If the target value is 1, which is AD, the function works vice versa. Thus, the weight parameters are updated to minimize the loss:

2 $$ {\text{loss}} = - {\text{t}}\log \left( {f\left( s \right)} \right) - \left( {1 - t} \right){ \log }\left( {1 - f\left( s \right)} \right). $$

The proposed network was trained to distinguish AD and NC in FDG-PET images using transfer learning. Transfer learning is a training method that updates the weight parameters. This enables the trained network of one specific domain to be applied in another domain [30]. We applied our model with the ImageNet and ADNI datasets for the transfer learning process. We first pretrained the entire network using the ImageNet dataset. Then, we fine-tuned the network using the ADNI dataset except at the first, second, and third convolution layers. This process enabled us to train a network that could classify AD and NC using 1245 PET images.

### Statistical analysis

We calculated the accuracy, sensitivity, and specificity of ADNI (which was not used for training) and of our dataset. To compare the performance using different datasets acquired under different protocols, we performed Pearson’s Chi-square test to show that there were no significant differences in performance between the datasets using our proposed method. We rejected the null hypothesis for *p* value < 0.05, to determine whether a difference was statistically significant. Moreover, we compared the model performance with and without the GAP layer. We used McNemar’s test to evaluate the presence of a statistical improvement using the GAP layer. We also rejected the null hypothesis for *p* value < 0.05. We implemented statistical analysis using Medcalc software.

## Data Availability

The ADNI datasets generated during the current study are available in the FDG-PET repository, https://ida.loni.usc.edu/.

## Abbreviations

AD: Alzheimer’s disease
FDG-PET/CT: F-18 Fluorodeoxyglucose positron emission tomography/computed tomography
NC: Normal cognitive
ADNI: Alzheimer’s disease neuroimaging initiative grand opportunities
GAP: Global average pooling
PCC: Posterior cingulate cortex
FDG: F-18 fluorodeoxyglucose
PET/CT: Positron emission tomography/computed tomography
CNN: Convolutional neural network
ILSVRC: Image-net large-scale visual recognition competition
FCN: Fully connected network
PVE: Partial volume effect
